# Hydrogels from TEMPO-Oxidized Nanofibrillated Cellulose Support *In Vitro* Cultivation of Encapsulated Human Mesenchymal Stem Cells

**DOI:** 10.1021/acsabm.2c00854

**Published:** 2023-02-06

**Authors:** Ilias Nikolits, Sara Radwan, Falk Liebner, Wolf Dietrich, Dominik Egger, Farhad Chariyev-Prinz, Cornelia Kasper

**Affiliations:** † Institute of Cell and Tissue Culture Technologies, Department of Biotechnology, University of Natural Resources and Life Sciences BOKU Vienna, Muthgasse 18, 1190 Vienna, Austria; ‡ Department of Life Science Engineering, University of Applied Sciences Technikum Vienna, Höchstädtplatz 6, 1200 Vienna, Austria; § Institute of Chemistry of Renewable Resources, Department of Chemistry, University of Natural Resources and Life Sciences BOKU Vienna, Konrad Lorenz Straße 24, 3430 Tulln, Austria; ∥ Department of Gynecology and Obstetrics, Karl Landsteiner University of Health Sciences, Alter Ziegelweg 10, 3430 Tulln, Austria

**Keywords:** mesenchymal stem cells, hydrogel, cellulose, 3D cultivation, *in vitro* culture

## Abstract

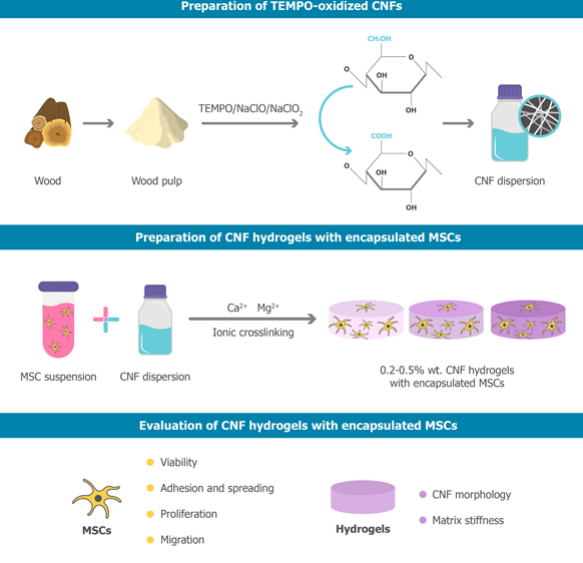

Mesenchymal stem cells (MSCs) are the most prominent type of adult stem cells for clinical applications. Three-dimensional (3D) cultivation of MSCs in biomimetic hydrogels provides a more physiologically relevant cultivation microenvironment for *in vitro* testing and modeling, thus overcoming the limitations of traditional planar cultivation methods. Cellulose nanofibers are an excellent candidate biomaterial for synthesis of hydrogels for this application, due to their biocompatibility, tunable properties, availability, and low cost. Herein, we demonstrate the capacity of hydrogels prepared from 2,2,6,6-tetramethylpiperidine-1-oxyl -oxidized and subsequently individualized cellulose-nanofibrils to support physiologically relevant 3D *in vitro* cultivation of human MSCs at low solid contents (0.2–0.5 wt %). Our results show that MSCs can spread, proliferate, and migrate inside the cellulose hydrogels, while the metabolic activity and proliferative capacity of the cells as well as their morphological characteristics benefit more in the lower bulk cellulose concentration hydrogels.

## 1 Introduction

The ability of mesenchymal stem cells (MSCs) to differentiate into multiple lineages^1^ as well as exert immunomodulatory properties^2^ has established this cell type as a prominent player in today’s field of regenerative medicine.^3^ Their plastic adherence^4^ and ready availability from various sources, including adipose tissue,^5^ bone marrow,^6^ and umbilical cord,^7^ further favored their employment in a considerable number of studies over the past years.^8,9^ However, there is an accumulating body of evidence indicating the negative impact of two-dimensional (2D) culture conditions on proliferation, differentiation, and immunoregulatory potential of MSCs,^10^ thus limiting the therapeutic potential of MSCs as well as the physiologic relevance and reproducibility of *in vitro* testing and modeling.^11^

In order to overcome limitations associated with 2D culture, 3D cell culture techniques have been developed in the last decades that aim to recapitulate aspects of *in vivo* microenvironment.^12−14^ Studies have shown that physiological functionalities and characteristics of MSCs can be maintained to a greater extent if cells are cultured in suitable 3D systems.^15−17^ The suitability of such systems is highly dependent on employed biomaterials and the associated biophysical properties, which ultimately affect adherence, proliferation, differentiation, and migration of MSCs.^18−20^ In this context, hydrogels have attracted attention in regenerative medicine due to their capacity to mimic aspects of the extracellular matrix (ECM) and thus provide a more *in vivo*-like microenvironment to the embedded cells during *in vitro* culture.^21^ Several types of synthetic and natural origin hydrogels, such as hyaluronic acid, alginate, collagen, gelatin, poly(ethylene glycol), and poly(lactic-*co*-glycolic acid) have been studied for cultivation of MSCs in 3D.^22^ Hydrogels for biomedical applications are often associated with certain drawbacks, such as low mechanical stability, high variability, use of chemical cross-linking agents or irradiation and manufacturing costs.^22,23^ A very promising candidate biomaterial is cellulose, a highly abundant and inexpensive natural organic polymer with broad utilization in a number of biomedical applications due to its excellent biocompatibility and tunable properties.^24^ Different types of nanocellulose have shown good biocompatibility with various types of human cells, such as fibroblasts,^25^ keratinocytes,^25^ and leukocytes.^26^ The anionic amphipathic nanofibrillar structure and bioinert nature of cellulose make it a highly suitable material for hydrogel development and 3D *in vitro* cultivation of cells.^24,27^ Furthermore, introduction of carboxylate groups into the native cellulose chains using established methods like 2,2,6,6-tetramethylpiperidine-1-oxyl (TEMPO) oxidation has been shown to facilitate nanofibrillation under shear force and increases the colloidal stability of respective aqueous dispersions at full preservation of its chemical integrity.^28^ Previous studies have examined *in vitro* the use of TEMPO-oxidized cellulose nanofibers (CNFs) as drug delivery systems,^29^ antimicrobial wound dressings, and tissue engineering constructs.^30^ Furthermore, pure and composite TEMPO-oxidized cellulose scaffolds have been shown to support *in vitro* cultivation of human MSCs cultured on the material surface.^31−33^ Although a small number of studies have investigated the capacity of hydrogels from TEMPO-oxidized CNFs to support *in vitro* cultivation of encapsulated human embryonic stem cells,^34,35^ their respective effects on human MSCs has not been investigated yet.

In this study, we prepared hydrogels and examined their mechanical and physical properties as well as suitability for human MSC *in vitro* culture applications. To this end, human adipose-derived MSCs were embedded in hydrogels with varying cellulose concentrations and were subsequently assessed for cell viability, metabolic activity, migration, proliferation, and morphology. We demonstrate that MSCs can spread, proliferate, and migrate inside all examined hydrogel compositions. Furthermore, we also demonstrate that higher cellulose concentrations directly affect morphology, metabolic capacity, and proliferation of embedded MSC.

## 2 Materials and Methods

If not stated otherwise, reagents were purchased from Sigma-Aldrich, St. Louis, MO, USA. All reagents and solutions were purchased at the highest available grade and were not further purified.

### 2.1 Cell Culture

The use of human tissue was approved by the ethics committee of Scientific Integrity and Ethics of the Karl Landsteiner University of Health Sciences (EK no. 1014/2019), and all donors gave written consent. Human adipose-derived MSCs (adMSCs) were isolated from skin flaps removed during routine relaparotomies, e.g., caesarian sections of female donors. The donor tissue was stored at 4 °C and processed within 24 h after the surgery. Briefly, fat tissue was minced with scissors, and digested with collagenase type IA for 1 h at 37 °C. Subsequently, a series of centrifugation and washing steps were performed to obtain the stromal vascular fraction, which was finally transferred in cell culture flasks and cultivated in standard expansion medium composed of MEM alpha (Thermo Fisher Scientific, Waltham, MA, USA), 0.5% gentamycin (Lonza, Basel, Switzerland), 2.5% human platelet lysate (hPL, PL BioScience, Aachen, Germany), and 1 U/mL heparin (PL BioScience, Aachen, Germany) in a humidified incubator at 37 °C and 5% CO_2_. When adMSCs reached approximately 80% confluency, they were detached using accutase (GE healthcare, Little Chalfont, UK) and cryo-preserved in MEM alpha, 2.5% hPL, 10% DMSO (Sigma-Aldrich, St. Louis, MO, USA), and 1 U/mL heparin in liquid nitrogen. After thawing, the cells were expanded for up to three passages in cell culture flasks (Sarstedt, Nümbrecht, Germany) and harvested by accutase treatment.

### 2.2 Preparation of TEMPO-Oxidized CNFs

TEMPO-oxidized CNFs were prepared as described previously.^36^ For cellulose starting material, never-dried bisulfite hardwood dissolving pulp was used. In brief, 40 g (dry weight) of the cellulosic material (50% water content) was suspended in 1.8 L of deionized water and disintegrated using a household blender for 1 min. Next, 640 mg of 2,2,6,6-tetramethylpiperidine-1-oxyl (TEMPO) (0.1 mmol/g of cellulose) and 4 g of NaBr (1 mmol/g of cellulose) were added to selectively oxidize the primary hydroxyl groups (C6) of cellulose. 60 mL of NaClO solution (available chlorine 10–15%) was added gradually to the mixture at room temperature, under constant stirring (1000 rpm) and with continuous pH adjustment to 10 by addition of 0.1 M NaOH. After the addition of NaClO, the oxidized cellulose was given 30 min of reaction time under constant stirring before it was washed with DI water and filtered multiple times. The oxidized pulp was then postoxidized by suspending the material in 1 L of 0.1 M CH_3_COOH solution and adding 9.72 g NaClO_2_ (2.7 mmol/g of cellulose), under constant stirring (1000 rpm) at room temperature overnight, in order to convert remaining intermediary aldehyde groups into carboxyl moieties. Following chlorite oxidation, and multiple washing steps by vacuum-aided filtration, the obtained 6-carboxyl cellulose was disintegrated in water to give a 0.5% w/w suspension. After adjusting the pH 8 by addition of diluted NaOH, the aqueous suspension was subjected to repeated (eight passes) of mechanical defibrillation at 80 MPa using a benchtop homogenizer (APV 1000, AxFlow GmbH, Premstätten, Austria). Aiming to reduce viscosity (and hence heating during nanofibrillation), the dispersion was diluted to 0.125% for the last three passes.

After homogenization, the dispersion of negatively charged cellulose nanofibrils was up-concentrated to a final concentration of 0.7 wt % using a rotary evaporator (40 °C, 45 mbar), and the pH was adjusted to 7.

### 2.3 Chemical and Morphological Characterization of CNFs

Analysis of the average carboxyl group content was performed by conductometric titration.^37^ An aliquot (52.25 mL corresponding to 65 mg CNF) of the 0.125 wt % CNF dispersion obtained after high-pressure homogenization was transferred into an Erlenmeyer flask, and 2.75 mL of 0.1 M hydrochloric acid was added. After that, conductometric titration was conducted by adding 25 μL aliquots of an aqueous solution of 0.1 M NaOH under continued stirring every 30 s using an automated titration unit (800 Dosino, 856 conductivity module Metrohm, Herisau, Switzerland). Evaluation of the titration curve, calculation of the degree of oxidation (DO), and determination of the carboxyl group content were accomplished as described elsewhere.^38^

Atomic force microscopy (AFM) was employed to visualize the prepared CNFs following the TEMPO-oxidation process described in Section 2.2 and assess their characteristics. The final oxidized CNF dispersion (0.7% wt.) was diluted 1:1000 in ultrapure Millipore grade water and dried on a mica plate. For the subsequent analysis by AFM, a Dimension Icon scanning probe microscope (Bruker, Santa Barbara, CA, USA) equipped with ScanAsyst-Air cantilever in tapping mode was used together with a NanoScope V control station. Gwyddion 2.40 software was used for image processing.

### 2.4 Preparation of CNF Hydrogels with Encapsulated adMSCs

Prior to encapsulation in the CNF hydrogels, cells were cultured as described in Section 2.1. After harvesting the cells by accutase treatment, they were resuspended in an appropriate volume of standard expansion medium (Section 2.1) for encapsulation in the hydrogels. Final aqueous CNF dispersion (0.7% wt.) was mixed with cell suspension using a Vortex-Genie 2 vortex mixer (Scientific Industries, Bohemia, NY, USA) at different mass ratios to achieve different bulk content concentrations of CNFs in the final hydrogels (0.2%, 0.3%, 0.4%, and 0.5% wt.). After mixing together the CNF dispersion with the cell suspension, the material was centrifuged for 3 min at 500*g* to remove air bubbles formed during mixing of the 2 phases and incubated at 37 °C and 5% CO_2_ for 30 min. Following incubation, the material was casted in 250 μL volume samples in 48-well plates, using a syringe and topped with 0.5 mL of standard expansion medium. Samples were placed on an orbital shaker at 100 rpm during the cultivation in an incubator at 37 °C and 5% CO_2_. The welled plates were coated with agarose to avoid background noise in the analyses from cells growing on the tissue culture-treated surface of the wells. Samples were prepared with 2.5 × 10^5^ cells/mL of hydrogel. Hydrogels without cells served as controls. Media change was performed every 2–3 days.

### 2.5 Live/Dead Cell Staining

Samples were stained with 1 μg/mL calcein AM (Invitrogen, Thermo Fisher Scientific, Waltham, MA, USA) and 3.3 μg/mL propidium iodide (Invitrogen) in phosphate-buffer saline solution (PBS), to visualize live and dead cells. Samples were incubated for 30 min at 37 °C in the dark, washed twice with PBS, and transferred to fresh standard expansion medium for fluorescence microscopic analysis.

### 2.6 Metabolic Activity

The metabolic activity of the hydrogel samples was measured using a resazurin-based viability assay (*In Vitro* Toxicology Assay Kit; TOX8), according to the manufacturer’s instructions. After removing the supernatant from the cultures, the same volume of standard expansion medium with 10% TOX8 was added. Hydrogels without cells were used as blank controls. The welled plates were incubated for 3 h on a shaker (100 rpm) at 37 °C and 5% CO_2_. After incubation, the fluorescence intensity of the supernatant was measured at 590 nm with an excitation wavelength at 560 nm, using an Infinite M1000 plate reader (Tecan, Männedorf, Switzerland). Measured values were normalized to blank controls.

### 2.7 Cell Morphology Staining

After cultivation, samples were fixed in 4% paraformaldehyde for 45 min and permeabilized with 0.5% Triton-X in PBS for 30 min prior to staining with the phalloidin staining solution. Samples were stained with 1 μg/mL Phalloidin iFluor-555 (Abcam, Cambridge, United Kingdom) in PBS with 1% BSA, to visualize the cytoskeleton arrangement of the hydrogel encapsulated MSCs by staining of the actin filaments. Samples were then incubated for 30 min at 37 °C in the dark, washed with PBS, and then inspected using a DM IL LED fluorescence microscope (Leica, Wetzlar, Germany).

### 2.8 EdU Assay

Detection of cell proliferation in the hydrogels was performed using EdU staining (EdU Click 488, baseclick GmbH, Munich, Germany). Hydrogel samples encapsulated with 5 × 10^5^ cells/mL of hydrogel were incubated with EdU at a concentration of 10 μM in cultivation media. After cultivation, samples were fixed in 4% paraformaldehyde for 45 min and permeabilized with 0.5% Triton-X in PBS for 30 min. The EdU staining procedure was performed according to the manufacturer’s instructions.

### 2.9 Migration Assay

adMSC spheroids of 2 × 10^5^ cells were formed in 96-well cell-repellent U-bottom microwell plates (Greiner Bio-One, Kremsmünster, Austria) for 3 days. Subsequently, the spheroids were encapsulated in 200 μL of CNF hydrogels of different concentrations (0.2%, 0.3%, 0.4% and 0.5% wt.) in 48-well tissue-culture treated plates. Calcein AM staining (Section 2.9) was used to visualize the migratory capacity of the cells from the initial spheroid mass into the hydrogel mass, overtime.

Quantification of the extent of migration of the cells in the hydrogels was performed based on the calcein AM fluorescence-stained area of the images, using CellProfiler 4.2.1 image processing software.^39^

### 2.10 Rheological Characterization of Hydrogels

Hydrogels of different concentrations (0.3%, 0.4%, and 0.5%wt) were prepared according to Section 2.4. In short, after mixing the cellulose dispersion with the cell suspension, disk-shaped hydrogels with approximately 2 cm diameter and 1.5 mm height were casted in Petri dishes and covered with standard expansion medium. Human adMSCs were encapsulated in those hydrogels, and their rheological properties were characterized. Hydrogels with adMSCs were compared to control hydrogels in the same medium but without cells.

Rheological measurements were conducted with an Anton Paar 302 Rheometer (MCR 302, Anton Paar GmbH, Austria). A parallel plate geometry (PP25) with a parallel surface was used. The gap size was set to 1.5 mm, based on achievable hydrogel thickness and minimal applied deformation due to sample loading and compression. After loading the cylindrical hydrogel samples, the samples were trimmed, and a resting time of 1 min was used to allow structural relaxation of the hydrogels before starting the measurement. The measurements were conducted at 37 °C; samples and geometry were equilibrated to this temperature, prior to measurements.

The rheological characteristics of hydrogels with different bulk content concentrations were investigated using the amplitude sweep method. Hydrogels of different concentrations with and without encapsulated adMSCs were prepared in disc shapes of 2 cm diameter and 1.5 mm height and tested on the parallel plate of an Anton Paar MCR 302 rheometer, under loading conditions/rate at 37 °C. The storage modulus (*G*′) of all samples was estimated within the linear elastic strain range at 0.5% shear strain.

Small amplitude oscillatory shear (SAOS) measurements were used to determine the viscoelastic properties of the hydrogels in the linear viscoelastic regime. To determine the elastic modulus *G*′ and viscous modulus *G*″, an amplitude sweep was performed with a shear strain range of 0.01–100%, with a logarithmic ramp. The test was performed at a frequency of 10/s. The elastic modulus was determined at a strain of 0.5%, which was within the linear viscoelastic regime for all samples (see Figure S2).

### 2.11 Statistical Analysis

All results are presented as mean ± standard deviation of at least three independent replicates. Two-way ANOVA followed by Tukey’s multiple comparisons test was performed, and data were plotted using GraphPad Prism 9.0.0 for Windows (GraphPad Software, San Diego, CA, USA). Significance is indicated as follows: * *p* < 0.05, ** *p* < 0.01, *** *p* < 0.001, and **** *p* < 0.0001.

## 3 Results

### 3.1 CNF Characterization and Hydrogel Preparation

Selective oxidation of the primary hydroxyl groups of cellulose was accomplished using the classical two-step alkaline route comprising: (1) TEMPO-mediated oxidation with sodium hypochlorite and (2) sodium chlorite treatment for onward oxidation of potentially formed carbonyl moieties. Subsequent high-pressure fibrillation in a dilute suspension state afforded a homogeneous dispersion of CNF that had an average carboxyl group content of 1.3 mmol/g, equivalent to a DO of 21%. Considering the chosen oxidation time and reactant proportions, these values are in good agreement with other studies.^40^

**Figure 1:**
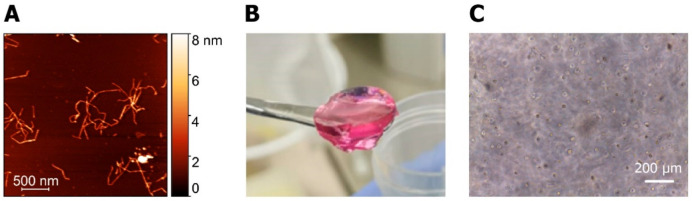
(A) AFM images of TEMPO-oxidized CNF shape and size. (B) Representative image of CNF hydrogels with encapsulated MSCs prepared in 48 well-plates. (C) Representative brightfield images of MSCs right after encapsulation in the CNF hydrogels.

Following preparation of the final cellulose dispersion (0.7 wt %) as described in Section 2.2, the shape and size of the resulting CNFs was examined. Obtained CNFs had an average length of 724 ± 256 nm (*n* = 20) and 3.3 ± 0.9 nm (*n* = 30) in width (based on fiber thickness and height measurements from extracted height profiles), as revealed by AFM image analysis (see Figure 1A). Final hydrogels were obtained by mixing the CNF dispersion with appropriate volume of cell suspension. Right after mixing the two phases, the mixture was easily casted in preferred shapes and volumes. After a 24 h incubation period for cross-linking between the CNFs, consistent volume hydrogels were obtained (see Figure 1B).

### 3.2 Viability and Metabolic Activity Evaluation of Encapsulated MSCs

**Figure 2:**
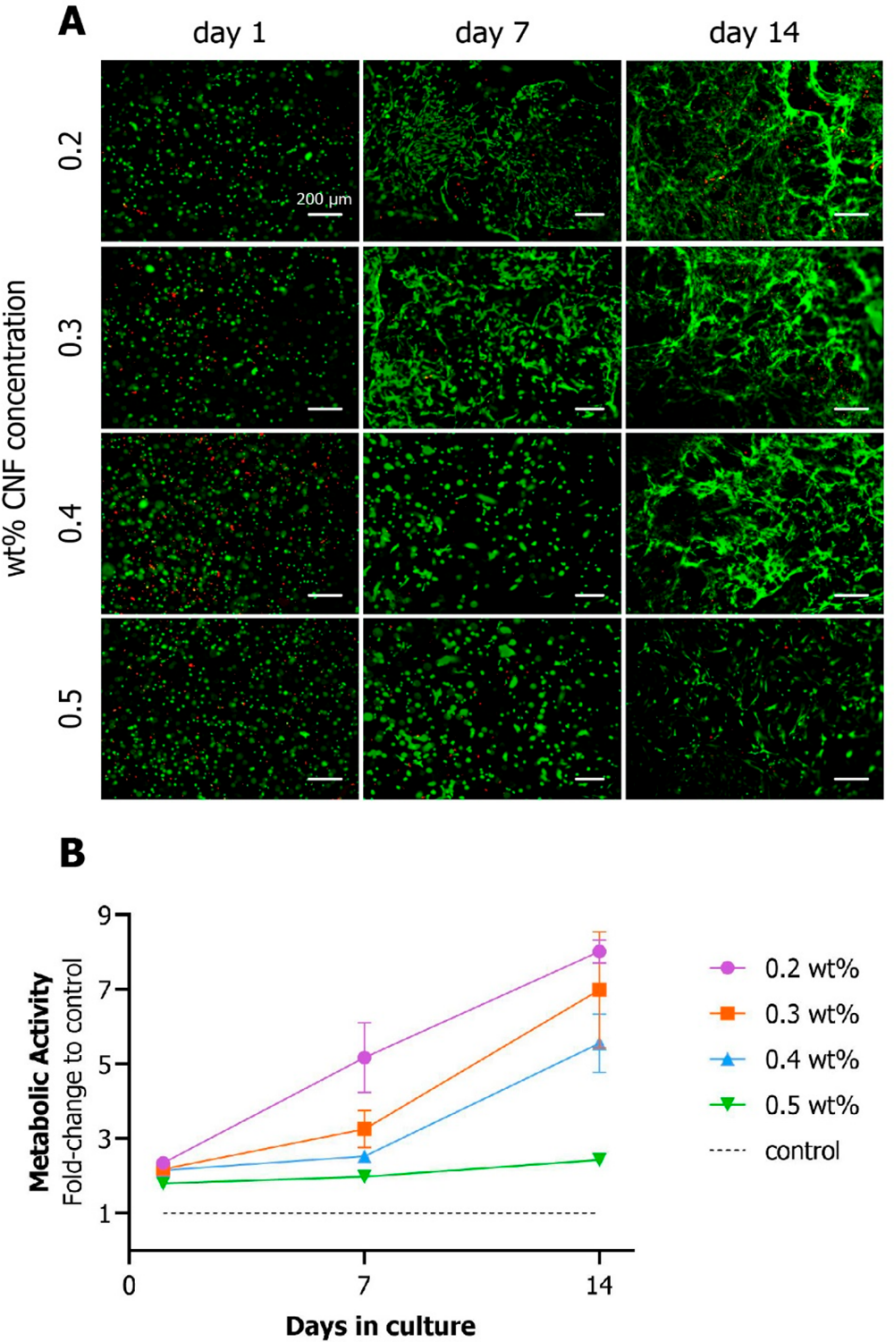
(A) Live/dead staining of encapsulated MSCs after 1, 7, and 14 days following encapsulation in CNF hydrogels with different wt % concentrations. Live cells were stained green (calcein AM), and dead cells were stained red (propidium iodide). Good viability was observed in all conditions at all time points. More pronounced cell spreading was visible in lower concentration samples. (B) Cell metabolic activity evaluation based on TOX-8 assay. MSCs encapsulated in CNF hydrogels showed increased metabolic activity overtime in comparison to control hydrogels samples without cells. Higher metabolic activity reported in samples with lower CNF concentrations.

To examine the capacity of hydrogels to support survival of MSCs, a live/dead staining and metabolic activity assay were performed on hydrogels prepared with different CNF concentrations (0.2–0.5 wt %) and cultured up to 14 days. The results of cell viability are presented in Figure 2A and reveal the presence of living cells across all examined hydrogel compositions and time points. Although a small number of dead cells was discernible after 24 h of encapsulation, the propidium iodide signal dissipated considerably at day 7 in all samples. On day 7, calcein AM intensity was higher in all conditions compared to day 1. Notably, the calcein AM signal further increased at day 14 in the 0.2, 0.3, and 0.4 wt % hydrogels, but there were no visible changes for 0.5 wt % samples. After 14 days in culture, a visible number of dead cells was detected in highly populated areas of 0.2 and 0.3 wt %. For the 0.4 and 0.5 wt % hydrogels, there was no visible difference in the number of dead cells between day 7 and day 14. Throughout the cultivation period, cells were evenly distributed in all examined hydrogel conditions. The results on the metabolic activity of encapsulated MSCs in different concentration hydrogels are in accordance with the live/dead fluorescence image analysis (see Figure 2B). Throughout the cultivation period, the metabolic activity increased continuously in all conditions. This increase was most evident in lower concentration hydrogels, thus revealing an inversely proportional relationship. In particular, the metabolic activity in all samples at day 1 was approximately 2-fold compared to the blank control and increased up to 8-, 7-, and 5.5-fold for the 0.2, 0.3, and 0.4 wt % samples at day 14, respectively. In contrast, the metabolic activity of 0.5 wt % samples increased only up to 2.5-fold at the end of the cultivation period.

### 3.3 Morphology of Encapsulated MSCs

**Figure 3:**
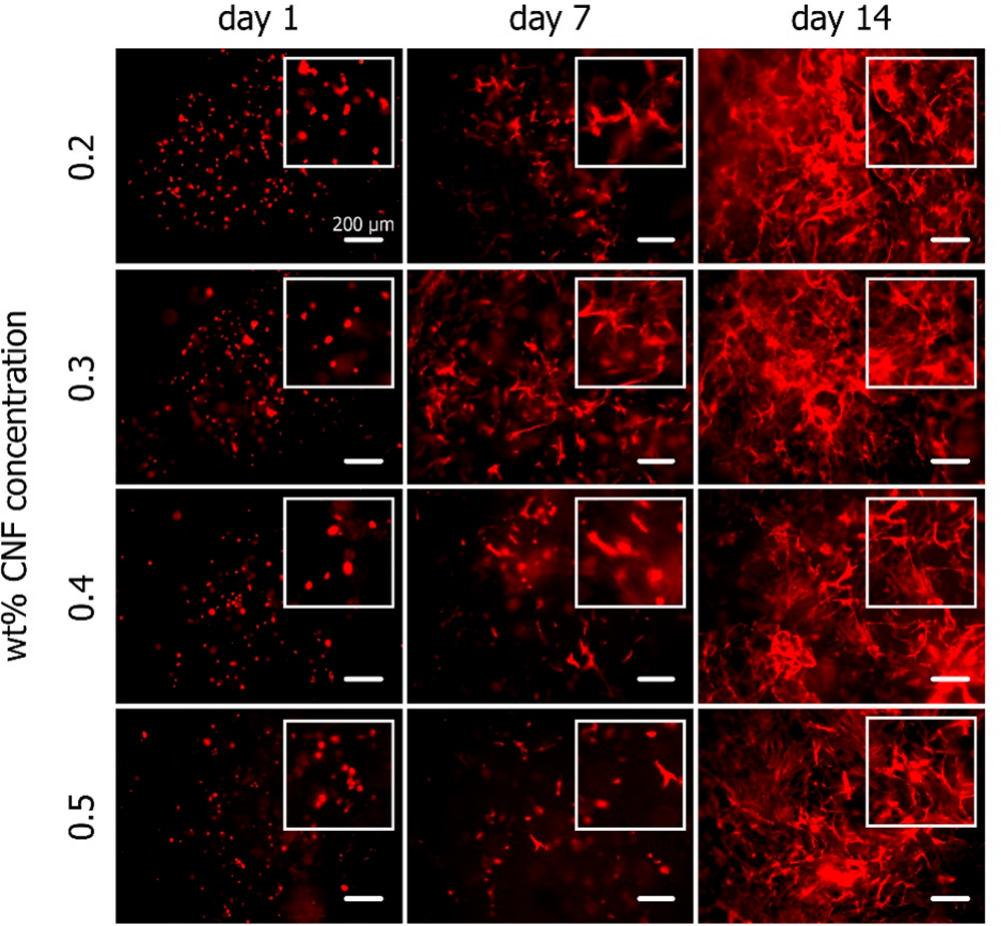
Cell morphology evaluation of MSCs embedded in CNF hydrogels of various concentrations. Actin filaments were visualized with phalloidin. Overtime, initially spherical MSCs stretched out into an elongated shape. The spindle-shaped morphology was more evident in earlier time points in the lower concentration samples.

An incremental spreading of cells was observed during viability assessment over the course of 14-day culture. In order to confirm this observation, an actin filament fluorescence staining assay was performed (Figure 3). Given enough time, all examined hydrogel concentrations allow spreading of human MSCs; however, lower concentration hydrogels enable faster morphological changes. Twenty-4 h after encapsulation, MSCs in all conditions remained spherical. After 7 days in culture, encapsulated cells in the 0.2 and 0.3 wt % started spreading in the typical for MSCs spindle-shaped morphology.^41^ In the 0.4 wt % samples, both slightly spread and still spherical-shaped cells were observed, while in the 0.5 wt % hydrogels, the majority of the cells remained spherical after 7 days. After 14 days, elongated spindle-shaped cells could be seen in all hydrogel conditions.

### 3.4 Proliferative Capacity of Encapsulated MSCs

**Figure 4:**
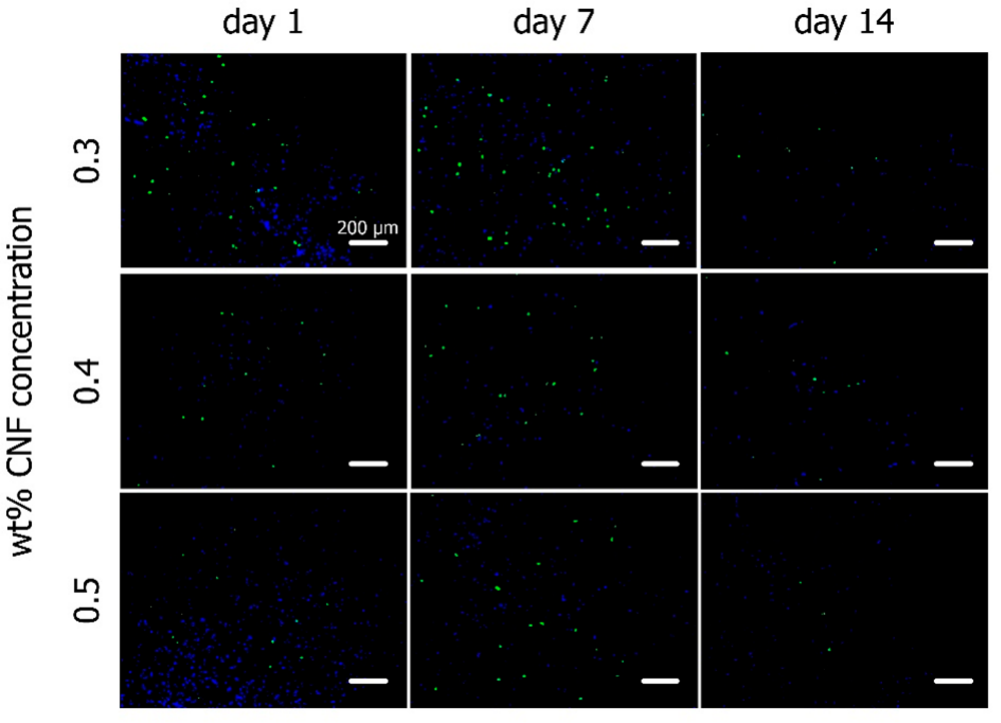
Cell proliferation evaluation based on EdU staining. All cell nuclei were stained blue (DAPI) and proliferating cell nuclei were stained green (EdU). An increased number of proliferating cells could be observed at day 7 in all hydrogel conditions. This effect diminished after 14 days in culture.

The effect of hydrogels with different CNF concentrations on the proliferative capacity of MSCs was investigated using an EdU *in vivo* cell proliferation kit. Due to dissociation of the 0.2 wt % samples during the staining process, this condition was exempt from this analysis. As seen in Figure 4, proliferating cells were visible in all examined hydrogel conditions 1 day following the encapsulation, while more dividing cells were visible in the 0.3 wt % samples. After 7 days, the number of dividing cells observed increased in all conditions. The proliferative capacity of the encapsulated cells seemed to have diminished after 14 days in culture in all hydrogels, as only a minimal number of EdU-stained cells were observed.

### 3.5 Migration Potential of Encapsulated MSCs

**Figure 5:**
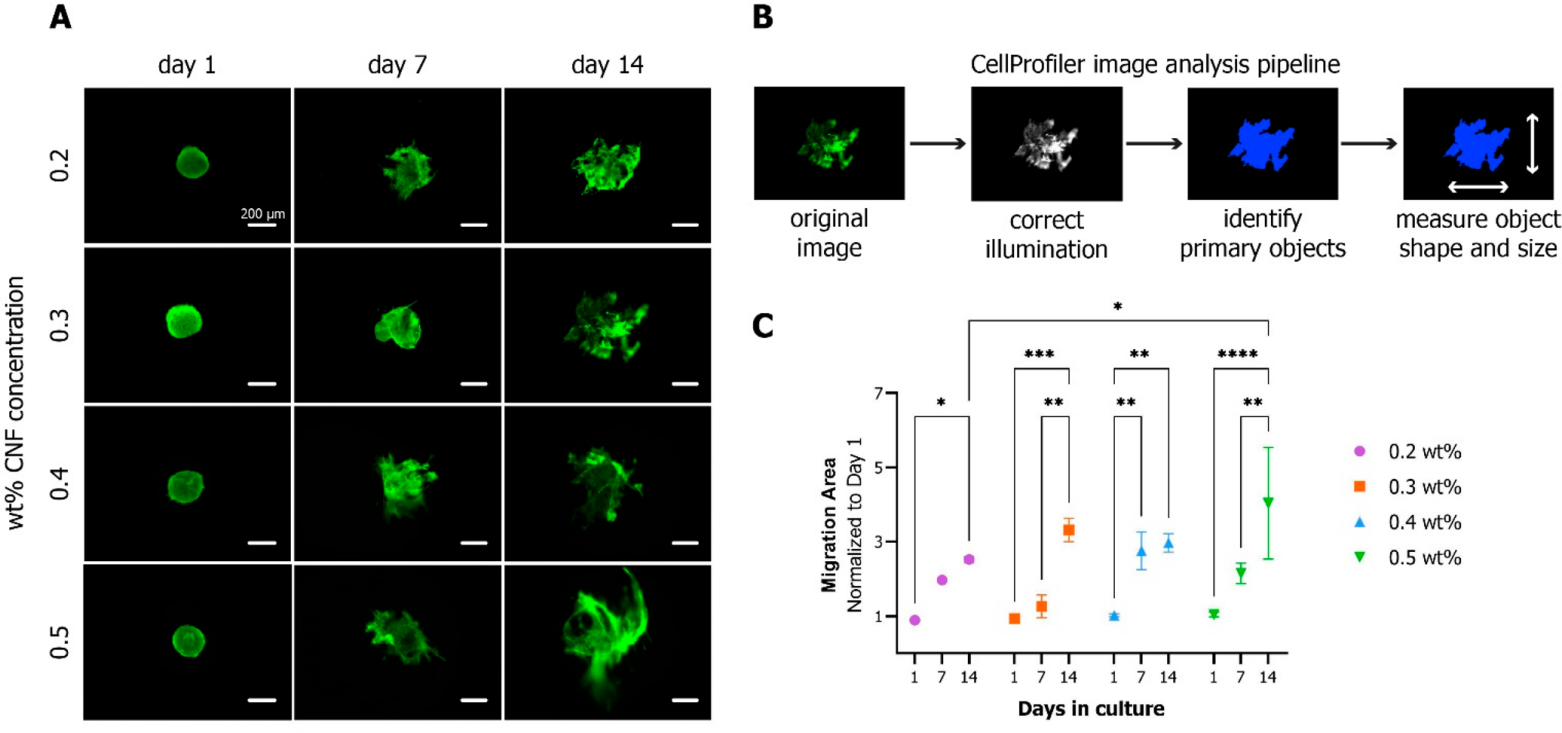
Cell migration evaluation based on calcein AM fluorescence-stained area. (A) MSC spheroids encapsulated in CNF hydrogels of different wt % concentrations migrated overtime from the initial spheroid mass into the hydrogel matrix. (B) Image analysis pipeline using CellProfiler software for calculating the fluorescence-stained area of the images. (C) Quantification of MSC migration capacity in the different hydrogel samples overtime, based on image analysis data. Significant increase of the extent of migration in all samples, relative to day 1 (*a* = 0.05).

The migration potential of MSC in the CNF hydrogels was evaluated by encapsulation of preformed cell spheroids in various concentration hydrogels, similarly to other studies.^42,43^ One day after encapsulation, the cell spheroids kept an intact spherical shape (see Figure 5A). After 7 days, spreading cells could be seen in all hydrogels, extending from the initial spheroids. This trend continued until day 14. The change in total area of the images covered by green (live) fluorescent cells was quantified using image analysis (see Figure 5B). The cell migration significantly increased over 14 days in all hydrogel concentrations (see Figure 5C) and was significantly higher in 0.5 wt % compared to 0.2 wt % hydrogels.

### 3.6 Mechanical Characterization of CNF Hydrogels

**Figure 6:**
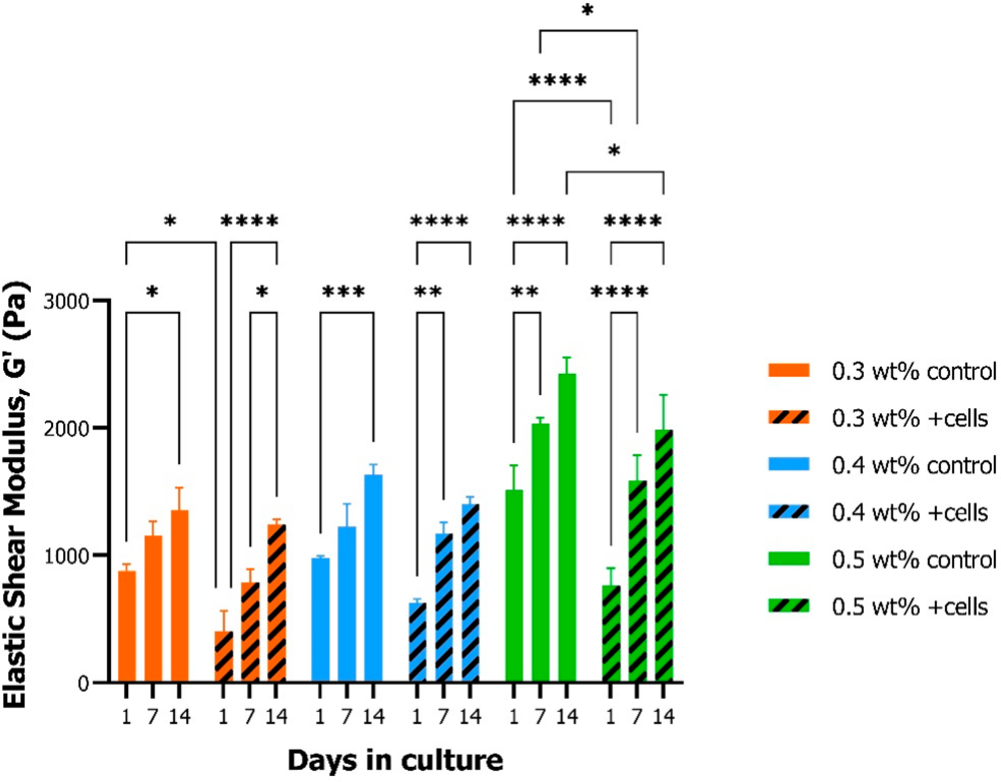
Mechanical characterization of CNF hydrogels. Elastic shear modulus measurements of different CNF concentration hydrogels with and without MSCs after 1, 7, and 14 days from cell encapsulation. Significant overtime stiffening was observed in all hydrogel sample conditions (*a* = 0.05).

The effects of CNF concentration and presence of cells on the mechanical properties of the hydrogels were evaluated by measuring the elastic shear modulus *G*′ by rheology. The preparation of 0.2 wt % CNF hydrogels with appropriate and consistent geometry for rheological analysis was not possible. Therefore, this condition was exempt from this analysis. Regardless of cell presence, *G*′ increased significantly over the course of 14 days in all hydrogel conditions (Figure 6). *G*′ of 0.5 wt % hydrogels was significantly higher compared to the 0.3 and 0.4 wt % samples with and without cells, respectively (Figure S1). 0.5 wt % control hydrogels exhibited significantly higher *G*′ values at all time points compared to the same concentration with encapsulated cells. The same trend was observed in the 0.3 and 0.4 wt % conditions, but with no statistical significance.

## 4 Discussion

Hydrogels represent an indispensable tool in today’s field of regenerative medicine due to their ability to provide physiologically relevant biological, mechanical, and chemical cues. In this study, cellulose-based hydrogels were prepared using different concentrations of TEMPO oxidized type I CNFs and used to encapsulate primary human adMSCs. The capacity of the hydrogels to support 3D cultivation of MSCs was evaluated by monitoring the viability, metabolic activity, morphology, proliferation, and migration of the cells over time.

An aqueous dispersion of individualized negatively charged CNFs was prepared by TEMPO-mediated oxidation of never-dried hardwood pulp in alkaline conditions and by subsequent mechanical disintegration using a high-pressure mechanical homogenizer, similar to what has been described before.^36,44^ Size and shape of obtained CNFs were in good agreement with the literature values of similar studies.^36,44^ The oxidation process introduces significant amounts of carboxylic groups into the native cellulose, without affecting their crystallinity and rod-like shape.^44^ The repulsive surface forces between the negatively charged oxidized CNFs facilitate individualization of the nanofibrils^45^ without affecting their particular cross-sectional (parallelogram) and longitudinal (right-handed twist) shapes.^28^ Due to mixing with cell culture media, consistent 3D hydrogel structures are formed by cross-linking of the CNFs. In particular, cations originating from the inorganic salts of the culture medium, e.g., CaCl_2_ and MgSO_4_, trigger physical cross-linking between the negatively charged carboxylate functional groups of the CNFs introduced by the TEMPO-mediated oxidation process.^29,46^ Hydrogels had to be incubated for at least 24 h after preparation to cross-link into consistent shapes and volume structures. For that reason, the first time point in all analyses was 1 day following preparation of the samples.

The bulk content concentration range (0.2–0.5%) of CNF hydrogels prepared in this study was in accordance to other CNF-based hydrogel studies with human stem cells.^34,35,47^ However, aforementioned studies never employed TEMPO-oxidized hydrogels in combination with human MSCs. Albeit commercially available CNF-based hydrogels were employed for culture of MSCs, their material formulations are nondisclosed. Therefore, to our knowledge there are no studies up-to-date to report the effects of TEMPO-oxidized CNF hydrogels on encapsulated human MSCs. Although a commercially available CNF-based hydrogel has been reported to support cultivation of human stem and progenitor cells with 1 wt % CNF concentration,^35^ the CNF hydrogel formulation investigated in the present study was not biocompatible for cultivation of encapsulated human MSCs in concentrations of 0.6 wt % or higher (data not shown). All different hydrogel formulations reported in our study supported 3D *in vitro* cultivation of primary human adMSCs; however, survival, metabolic activity, and proliferation rates were inversely proportional to the CNF concentration of hydrogels. A visible number of dead cells in the samples the day following encapsulation might have been a result of the stress experienced by the cells during the hydrogel encapsulation process. Furthermore, the number of dead cells in the highly populated parts of the hydrogels at the late time points is similarly reported in planar MSC cultures, with increasing numbers of dead cells when the culture surface becomes overconfluent with cells.^48^ Lower concentration hydrogels appeared to better support *in vitro* cultivation of MSCs as the metabolic activity and the number of viable and proliferating cells increased overtime. Similar results relative to the CNF concentration of the hydrogels were observed in a similar study using market-scale CNF-based hydrogels with nondisclosed material formulations.^47^ The different CNF concentrations in the prepared hydrogels in the present study resulted in softer and stiffer hydrogels, as confirmed by the mechanical assessment of the samples. It has been well established that matrix stiffness significantly impacts the biological behavior and characteristics of MSCs during 3D *in vitro* cultivation with hydrogels.^49^ However, the stiffness of different hydrogel systems should be compared with care as the impact of stiffness is a result from hydrogel properties such as type and synthesis of the hydrogel material, cross-linking, molecular weights, and proportion of components, and thus cannot be decoupled from them.^50^

Aggregation is a phenomenon often noticed during hydrogel encapsulation of single cells^51^ which can limit the viability of the 3D culture due to the formation of necrotic cores in the aggregates.^52^ In the presented encapsulation approach, an even distribution of cells was observed in all different concentration hydrogels without any signs of aggregation.

Interestingly, encapsulated MSCs were able to progressively spread into connected spindle-shaped morphologies overtime, similar to what has been reported in other types of hydrogels.^42^ This morphological effect was also inversely proportional to the CNF concentration of the hydrogels. The hydrophilic domains^53^ together with the specialized binding domains of cellulose allow MSC to attach and spread^54^ along the nanofibrillar network of the hydrogels during encapsulation. To that end, the material does not require any peptide sequence surface modifications to promote cell adhesion,^55^ such as incorporation of the arginine-glycine-aspartate (RGD) which has been commonly used to enhance biomaterial cell adhesion properties.^56,57^ This adhesion and spreading of MSCs on the CNFs was also reported in another study using a commercial CNF based hydrogel,^58^ but in other studies using human mesenchymal^47^ and murine embryonic stem cells,^34,59^ encapsulated in different types of CNF-based hydrogels, it was not observed. 3D-matrix adhesion patterns are different from the 2D *in vitro* substrate adhesions in terms of localization, function, and structure and therefore can potentially be more physiologically relevant.^60^

*In vivo* MSCs migrate via blood circulation into injured tissue sites, stimulated mainly by immunomodulatory factors and contribute in tissue repair and regeneration.^61^ Many regenerative therapy strategies are based on this intrinsic property of MSCs.^61^ Therefore, the migratory capacity of MSCs was evaluated in the different CNF concentration hydrogels. In line with the literature, matrix stiffness differences affect MSC migration and adhesion capacity.^62^ Stiff matrix hydrogels have been noted to promote MSC migration better than soft ones,^63^ which in the present study was only significantly different between the 0.2 and 0.5 wt % conditions after 14 days in culture. The stiffness differences between the CNF hydrogel conditions tested in this study (<2-fold) were not that large compared to the stiffness value ranges tested in the literature reports (3–20-fold), and that could explain why no significant differences were observed between the reported conditions.

Mechanical assessment of the hydrogels confirmed an expected effect on the stiffness of the hydrogels relative to their CNF concentration. Higher CNF contents in the hydrogels enhances their stiffness due to higher number of CNFs reinforcing the matrix and the enhanced cross-linking opportunities provided by the higher count of available surface- functional groups.^64^ However, the same analysis also revealed an increase of *G*′ of the hydrogels throughout the entire cultivation time of 14 days. This can be explained by progressing cross-linking of surface carboxylate groups governed by the accumulating quantity of cations added during media changes. Addition of cells in the hydrogels resulted in a decrease in stiffness in all sample conditions over time, similar to what has been reported in other types of hydrogels following cell encapsulation.^65^ A plausible explanation for the *G*′ decrease would be that the presence of the softer than the hydrogel cells contribute to the overall stiffness of the samples. The minimum *G*′ value measured for the control hydrogels was close to 0.9 kPa, while the elastic modulus of single stem cells is around 0.5 kPa or lower.^66^ Another potential reason for the hydrogel softening is that the presence of cells in the matrix impedes the cross-linking between the available functional groups of the CNFs, similarly to what was described in another study.^67^ The elastic shear moduli of all control hydrogels were in the physiological range of reported values (0.5–7.5 kPa) from empirical^68^ and theoretical^69,70^ subcutaneous human and porcine adipose tissue measurements, therefore increasing their physiological relevance as 3D *in vitro* cultivation platforms.

## 5 Conclusions

In summary, we performed a comprehensive analysis of a novel application of TEMPO-oxidized CNF-based hydrogels for physiologic 3D *in vitro* cultivation of encapsulated human MSCs. Our focus was on the use of widely available, low-cost, and biocompatible material, previously used for biomaterial and tissue engineering applications but not with the specific use and effects on MSCs. The main conclusion of this study is that the hydrogels from TEMPO-oxidized cellulose with low CNF concentrations (0.2–0.5 wt %) are an effective platform for physiologic 3D cultivation of encapsulated human MSCs by supporting (1) survival, (2) proliferation, (3) migration, and (4) adhesion and spreading of the cells *in vitro*. Therefore, these hydrogels represent a very promising biomaterial platform for more physiological 3D cultivation of human MSCs for *in vitro* testing and modeling and with prospects for clinical applications.

## Supporting Information Available

Additional statistical analysis data on mechanical characterization of CNF hydrogels and SAOS measurement graphs for all tested conditions (PDF)
