# Supplementary material for: Hydrogels from TEMPO-Oxidized Nanofibrillated Cellulose Support In Vitro Cultivation of Encapsulated Human Mesenchymal Stem Cells
Source: ACS Appl Bio Mater. 2023 Feb 6;6(2):543–51. doi: 10.1021/acsabm.2c00854 (PMC9945099; doi:10.1021/acsabm.2c00854)
Supplement: Supplementary file 1 — mt2c00854_si_001.pdf [file mt2c00854_si_001.pdf]

Supporting Information

Hydrogels from TEMPO-oxidized nanofibrillated cellulose support *in vitro* cultivation of encapsulated human mesenchymal stem cells

*Ilias Nikolits [1], Sara Radwan [2], Falk Liebner [3], Wolf Dietrich [4], Dominik Egger [1], Farhad Chariyev-Prinz [1] and Cornelia Kasper [1, *]*

[1] Institute of Cell and Tissue Culture Technologies, Department of Biotechnology, University of Natural Resources and Life Sciences BOKU Vienna, Muthgasse 18, 1190 Vienna, Austria

[2] Department of Life Science Engineering, University of Applied Sciences Technikum Vienna, Höchstädtplatz 6, 1200 Vienna, Austria

[3] Institute of Chemistry of Renewable Resources, Department of Chemistry, University of Natural Resources and Life Sciences BOKU Vienna, Konrad Lorenz Straße 24, 3430 Tulln, Austria

[4] Department of Gynecology and Obstetrics, Karl Landsteiner University of Health Sciences,

Alter Ziegelweg 10, 3430 Tulln, Austria

Corresponding Author: *Cornelia Kasper**.** Email: cornelia.kasper@boku.ac.at

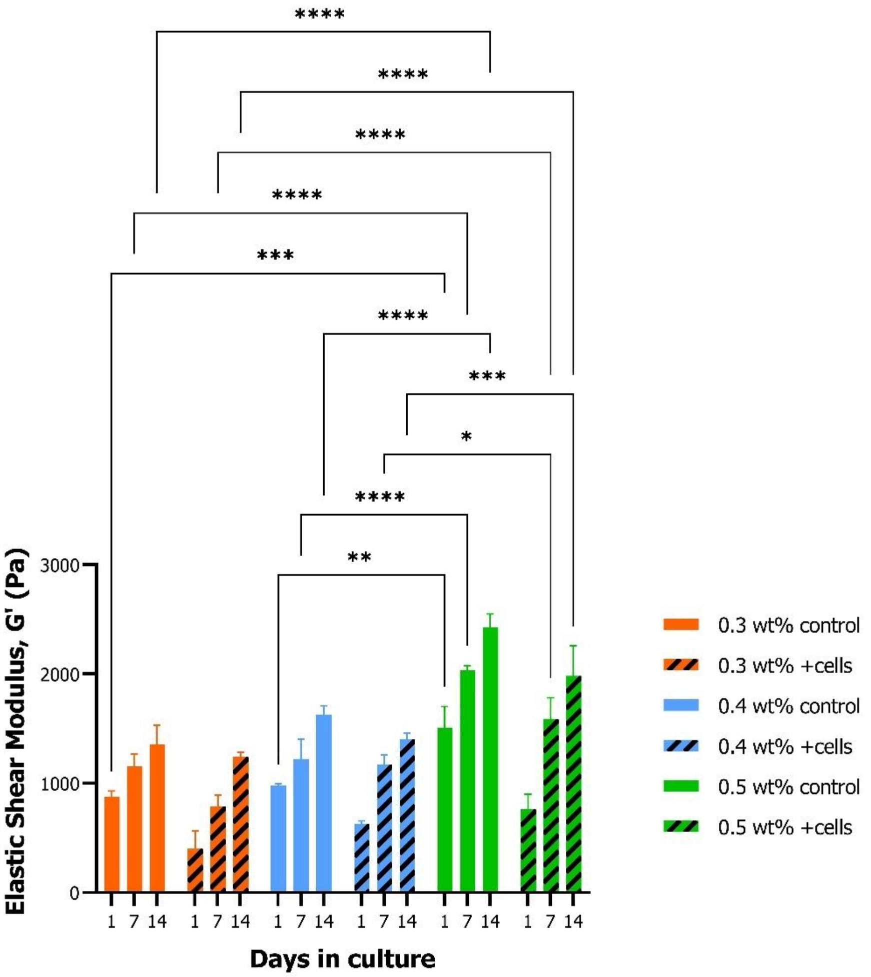

Figure S1. Mechanical characterization of CNF hydrogels. Elastic shear modulus measurements for different CNF concentration hydrogels with and without MSCs after 1, 7 and 14 days from cell

encapsulation. Significantly higher G’ in relation to the CNF wt% concentration of the samples at different time points of culture (a=0.05).

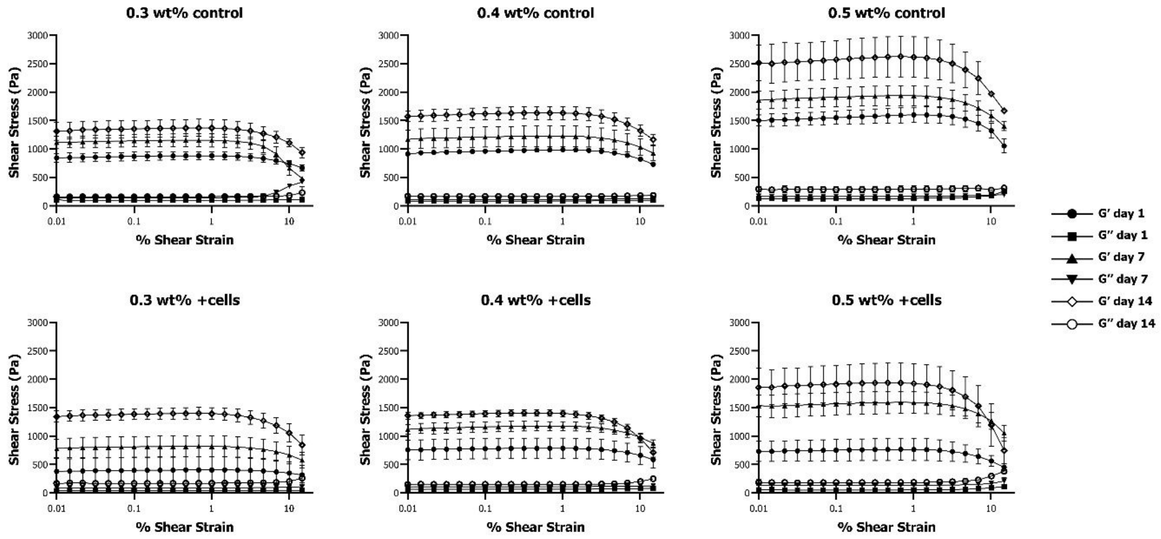

Figure S2. SAOS measurements performed to evaluate the viscoelastic properties of the different CNF concentration hydrogels with and without MSCs after 1, 7 and 14 days from cell encapsulation. The elastic shear modulus G’ was determined at a strain of 0.5%, which was within the linear viscoelastic regime for all samples.
